# The effect of dexmedetomidine on the perioperative hemodynamics and postoperative cognitive function of elderly patients with hypertension: Study protocol for a randomized controlled trial

**DOI:** 10.1097/MD.0000000000012851

**Published:** 2018-10-26

**Authors:** Xuejiang Du, Jianshe Yu, Weidong Mi

**Affiliations:** a Department of Anesthesiology, Chinese PLA Medical School/Chinese PLA General Hospital, Beijing; b Department of Anesthesiology, The Affiliated Hospital of Inner Mongolia Medical University, Hohhot, China.

**Keywords:** dexmedetomidine, elderly with hypertension, myocardial injury, postoperative cognitive function

## Abstract

**Introduction:**

Cognitive dysfunction after surgery, a common clinical manifestation of postoperative psychonosema. It usually occurs after heart surgery, hip replacement, mandibular fractures, and other major operations. Dexmedetomidine can exert sedative, analgesic, anxiolytic effect, inhibits the sympathetic activity, maintains hemodynamic balance, helps reduce the amount of anesthetic agents, and relatively slightly depresses respiration. Preoperative administration of dexmedetomidine for sedation has been reported to reduce the incidence of acute postoperative delirium. But currently there is no study on the effect of dexmedetomidine on the postoperative cognitive function of elderly patients with essential hypertension.

**Methods/Design:**

This study is a prospective, single-center, double-blind controlled clinical trial. Elderly patients aged between 60 and 80 years old, diagnosed with primary hypertension for 1 year or longer will be included, and randomized into 2 groups. Patients in observational group will be given a loading dose of dexmedetomidine at 0.8 μg/kg, pumped for over 10 minutes. Although patients in control group will be pumped of the same volume of normal saline within 10 minutes, before the induction of anesthesia. Minimental state examination and levels of interleukin-6, tumor necrosis factor alpha, and C-reactive protein will be set as primary endpoints. Baseline characteristics of patients will be summarized by groups and compared using Chi-square or Fisher exact tests for categorical variables and 2-sample *t* tests or Wilcoxon rank sum test for the continuous variables. Repeated measurement analysis of covariance model will also be used for the comparison of endpoints between 2 groups.

**Conclusion:**

The present study is designed to investigate the effect of the application of dexmedetomidine on postoperative myocardial injury and postoperative cognitive dysfunction, also to explore the association between inflammatory factors and postoperative cognitive function. With this study, we are expecting to find out an appropriate anesthesia method for elderly people with hypertension to alleviate the postoperative adverse effects caused by medical treatments.

**Trials registration:** This study was registered on Chinese Clinical Trial Registry (http://www.chictr.org.cn/) with the ID ChiCTR-IPR-16009156.

## 1 Background

Dexmedetomidine is a novel highly selective α2-adrenoceptor agonist. The affinity of dexmedetomidine to α2-adrenergic receptor is 8 times higher than clonidine, another α2-adrenergic receptor agonist. The ratio of α2-adrenergic receptors to α1-adrenergic receptors binding with dexmedetomidine is 1620:1.^[1]^ Dexmedetomidine exerts sedative, analgesic, anxiolytic effect, inhibits the sympathetic activity, maintains hemodynamic balance, helps reduce the amount of anesthetic agents, and relatively slightly depresses respiration.^[2,3]^ In addition, it shows the antisialagogue, antishivering, and diuretic effects.^[4]^ In 1999, the drug was approved by US Food and Drug Administration (FDA) to be used for sedation in intensive care unit (ICU). In 2009, it was approved by FDA to be used for sedation on patients receiving endotracheal intubation and mechanical ventilation under general anesthesia. Currently, it is mainly applied for sedation in perioperative period and in ICU in China.

Slow pump administration of dexmedetomidine may decline its biphasic effect on blood pressure in a dose-dependent manner, which is also affected by the rate of administration. Rapid infusion of dexmedetomidine directly activates α2B-vasoconstrictor receptors and can cause transient high blood pressure, leading to reflexive heart rate reduction.^[5]^ Continuous infusion may produce antisympathetic effect and enhance vagal activity, decreasing blood pressure, and heart rate. Intravenous administration of dexmedetomidine before anesthesia can suppress hemodynamic fluctuation during intubation, and reduce the dosages of anesthetic drugs and opioids.^[6–10]^ In the study on nasotracheal intubation, dexmedetomidine was better tolerated compared with fentanyl or propofol. And patients receiving dexmedetomidine had more stable hemodynamics. In the myocardial ischemia model of rats in vivo, dexmedetomidine administered before ischemia and hypoxia can reduce coronary blood flow and myocardial infarction area, and improve the restoration of coronary blood flow.^[11]^ In healthy volunteers, the infusion of dexmedetomidine can reduce myocardial blood flow and decline myocardial oxygen demand. However, this does not cause myocardial ischemia. The prevalence of hypertension in elderly people is remaining at a high level of 60% to 80%, as shown by the previous surveys.^[12–15]^ It is an important population calling for concern. Thus it provokes our interest to investigate the effect of dexmedetomidine on the cardiac muscle of elderly patients with hypertension, because there are few studies focusing on this so far.

Dexmedetomidine decreases cerebral blood flow in a dose-dependent manner. However, the autoregulation of CO_2_ reactivity and cerebral vessels maintains. Animal experiments showed dexmedetomidine had a protective effect on brain. It acts on α2A receptors and reduces the area of grey and white matter lesions caused by perioperative excitotoxins. On rats with a lack of α2A receptors, dexmedetomidine cannot protect the white matter from injury. In addition, the white matter lesion is relatively serious. In terms of topical effects, after global cerebral ischemia and ischemia/reperfusion, dexmedetomidine can improve the survival rate of nerve cells. The mechanism of brain protection effects can be summarized as follows: reduction in the release of norepinephrine from brain tissue, regulation of the balance of apoptotic proteins and antiapoptotic proteins, reduction in the release of the excitatory neurotransmitters in the brain, the phosphorylation of heat shock protein 27 and the decreased expression of caspase-3 in brain tissue, the activation of extracellular signal regulated protein kinase 1/2 to produce neuroprotection.

Cognitive dysfunction after anesthesia, a common clinical manifestation of postoperative psychonosema, is gradually focused now.^[16,17]^ This usually occurs after heart surgery, hip replacement, mandibular fractures, and other major operations.^[18]^ The manifestations are disorder in memory, abstract thinking, and disorientation after anesthesia, meanwhile accompanied by decreased social activity (i.e., changes in personality, social skills, cognitive capabilities, and skills). Mild cases only present cognitive abnormalities. In moderate cases, the patients suffer severe memory loss, or amnestic syndrome. In severe conditions, the patients develop dementia with severe memory impairment, the loss of judgment and language summarization ability, as well as personality changes. Based on varying degrees of cognitive impairment, the conditions can be divided into 3 degrees: mild neurocognitive abnormalities, amnesia, and dementia. For mild cases, the condition has a short duration and can disappear spontaneously. Only life and work are troubled. However, moderate cognitive disorders such as loss of judgment and language summarization ability, personality changes, and severe conditions such as senile dementia, can lead to the decline or loss of social activities, work, and self-care ability. Increasing age, low education level, prolonged duration of anesthesia, a second surgery, postoperative infections, and respiratory complications all increase the risks of postoperative cognitive dysfunction (POCD).^[16,17]^ Minimental state examination (MMSE) is a commonly used neuropsychological scale in clinical practice.^[19]^

In previous study on patients receiving cardiothoracic surgery, the comparison of the sedation effect of postoperative dexmedetomidine, propofol, and midazolam showed the incidence of delirium in the dexmedetomidine group was 8%, but 50% in the other 2 groups. Moreover, it has been reported that preoperative administration of dexmedetomidine for sedation may also reduce the incidence of acute postoperative delirium.^[20]^ However, currently, no study on the effect of dexmedetomidine on the postoperative cognitive function of elderly patients with essential hypertension has been reported.

Interleukin-6 (IL-6), a proinflammatory cytokine which is mainly produced by monocytes, macrophages, and T cells, has a wide range of biological activities. Among proinflammatory cytokines, IL-6 is the most potent inflammatory mediator that endogenously promotes systemic inflammatory response. It is the major effector to produce acute phase proteins and gather inflammatory cells. Compared with IL-1β and tumor necrosis factor alpha (TNF-α), IL-6 is more directly related to operation. The increased amplitude and duration are roughly consistent with the severity of trauma. IL-6 is a sensitive marker of tissue damage, in particular, to indicate the severity of inflammatory response. Vascular wall thickening and endothelial cell damage lead to angiosclerosis and increasing resistance of peripheral vessels. This is an important etiology for the onset and development of primary hypertension. IL-6 and IL-8, as cytokines highly correlated with inflammation, play important roles in the pathogenesis of primary hypertension. TNF-α is one of the important mediators that appear earliest in the systemic inflammatory response caused by trauma and stress. TNF-α stimulates vascular endothelial cells to express adhesion molecules, facilitating its adherence to leukocytes, and stimulates monocytes macrophages and other cells to secrete chemokines, causing accumulation of leukocytes at sites of inflammation.^[21,22]^ Studies have reported significantly increasing serum C-reactive protein (CRP) in patients with hypertension, and the level of high-sensitivity C-reactive protein increases with the prolonged course of hypertension.^[23,24]^

Here, we present the study protocol for a random clinical trial to examine the effect of dexmedetomidine on the perioperative circulation and postoperative cognitive function of elderly patients with hypertension, to provide clinical reference for peers to give appropriate treatment regimen.

## 2 Method/design

### 2.1 Study design

**Figure 1:**
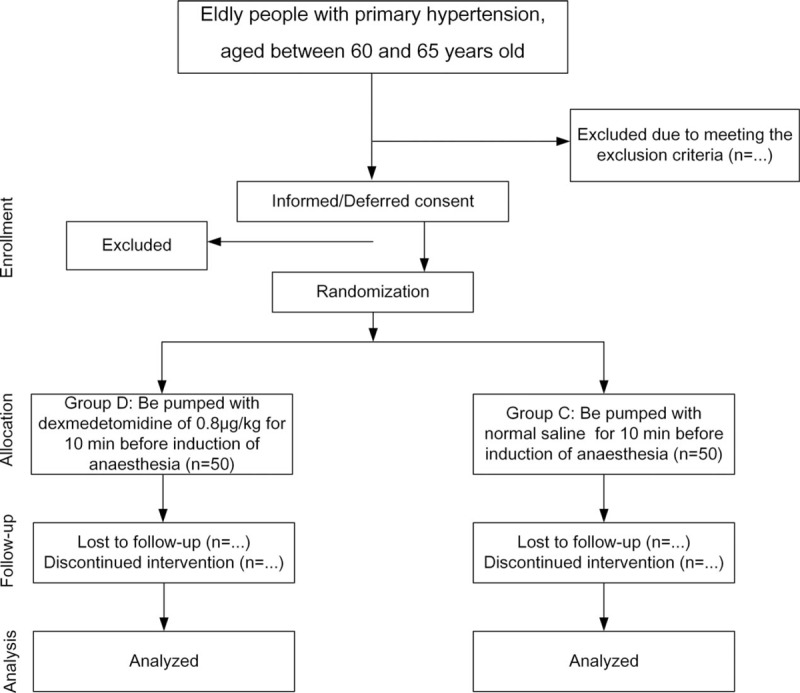
Study design and participant flow chart.

This study is a prospective, single-center, double-blinded controlled clinical trial to investigate the effect of dexmedetomidine on the perioperative circulation and postoperative cognitive function of elderly patients with hypertension (Fig. 1).

### 2.2 Ethics

This study has been approved by the ethics committee of First Affiliated Hospital of Mongolia Medical University, and registered on Chinese Clinical Trial Registry with the ID ChiCTR-IPR-16009156. Informed consents will be obtained from all the subjects involved.

### 2.3 Study population

#### 2.3.1 Inclusion criteria

1. Subjects, aged between 60 to 80 years old, diagnosed with primary hypertension for 1 year or longer (blood pressure ≥140/90), American Society of Anesthesiologists grade II or III, ready for elective gastrointestinal surgery.
2. Body weight between 45 and 75 kg, body mass index between 19 and 24 kg/m^2^.
3. The health conditions of the subjects evaluated to be generally well according the medical history, physical examination, and laboratory tests. No signs of difficult intubation. No obvious mental disorders.
4. Normal cognitive function, MMSE score >27 points.
5. Normal ST segment in preoperative electrocardiogram.

#### 2.3.2 Exclusion criteria

1. Significant arrhythmias, atrioventricular block, liver dysfunction, kidney dysfunction, pulmonary disease, endocrine disease, any other diseases or pathological conditions that can interfere with the experimental results.
2. Serious hearing, visual impairment, or other conditions that hamper communication with the subjects.
3. Long-term administration of sedative(s), hypnotic(s), and antidepressant(s).
4. A history of cerebral infarction, other central nervous system diseases, organic brain diseases, or mental illness.
5. Alcohol or drug addiction.
6. Known allergy to study drugs.
7. Secondary hypertension.

### 2.4 Study settings

The present study will be conducted in *the Affiliated Hospital of Inner Mongolia Medical University*, a tertiary hospital located in *Hohhot*, *Mongolia* province, China.

### 2.5 Study group

One hundred elderly hypertensive patients who meet the criteria and receive elective surgery will be selected. The subjects will be randomly divided into 2 groups, namely the dexmedetomidine group (group D) and control group (group C), 50 cases in each group.

### 2.6 Study time

This clinical trial will be conducted from September 5, 2016 to September 5, 2019.

### 2.7 Interventions

Monitoring will be exerted throughout the operation, including 5-lead electrocardiograph (ECG), ambulatory blood pressure, surplus pulse O_2_, the end-tidal CO_2_ pressures and bispectral index (BIS), on all the patients involved. Before the induction of anesthesia, patients in group D will be given a loading dose of dexmedetomidine at 0.8 μg/kg, pumped for over 10 minutes. Subsequently, dexmedetomidine will be continuously intravenously infused at the rate of 0.5 μg/kg · h. Patients in group C will be pumped of the same volume of normal saline within 10 minutes.

The induction of anesthesia will be performed as per the following method: 0.05 mg/kg midazolam, 4–5 μg/kg fentanyl, 1.0 to 1.5 mg/kg etomidate, and 0.15 mg/kg cis-atracurium besilate will be intravenously administrated on subjects in both groups. After endotracheal intubation, DrägerGS anesthesia machine will be connected, and the tidal volume will be set at 8 to 10 mL/kg, respiration rate at 10/min. By adjusting the respiratory parameters, the intraoperative the end-tidal CO_2_ pressures (P_ET_CO_2_) and BIS value will be maintained at 35 to 45 mm Hg and 40 to 60, respectively.

Anesthesia maintenance: continuous pump of cis-atracurium besilate 0.05 to 0.1 mg/kg · h, propofol 4 to 8 mg/kg · h, and remifentanil 0.1 to 0.2 μg/kg · min. After the operation, the patients will be transferred to postanesthesia care unit with the endotracheal tubes, which will not be removed until the patients meet the extubation criteria.

### 2.8 Adverse events observation

When intraoperative mean arterial pressure is 20% lower than the baseline blood pressure, infuse fluids rapidly. If it is not effective, intravenously inject ephedrine 5 mg. When the mean arterial pressure is 20% higher than the baseline blood pressure, intravenously administer urapidil 10 mg. When heart rate is <55 bpm, intravenously administer atropine 0.3 to 0.5 mg.

### 2.9 Primary outcomes

MMSE scores 1 day before and 3 and 6 days after the operation. The levels of IL-6, TNF-α, and CRP in peripheral blood before induction (T1), 6 hours after the end of the operation (T10) and 24 hours after the end of the operation (T12).

### 2.10 Secondary outcomes

Heart rate, systolic blood pressure, diastolic blood pressure, and ST-segment changes in ECG in the 2 groups before induction (T1), 3 minutes after induction (namely before intubation, T2), immediately after intubation (T3), 1 minute after intubation (T4), 3 minutes after intubation (T5), 5 minutes after intubation (T6), 1 hour after the start of the operation (T7), and at the end of the operation (T8).

### 2.11 Estimation of sample size

We calculate the sample size based on the primary outcome MMSE score. Our pilot study showed MMSE score post operation were different between the D and C groups a difference of 1.2 and standard deviation (SD) of MMSE score of 1.8 in each group. Based on a 2-sample *t* test, we will need 36 patients in each group to finish the study to detect such a difference with 80% power and 5% type I error rate. Assuming a 20% drop out rate, we will need 45 patients to start with. With 50 patients in each group, we will have >80% power. The analysis using repeated measurement analysis of covariance (ANCOVA) model will be more powerful.

### 2.12 Randomization and blinding

After recruitment, patients who meet the eligibility criteria will be randomly assigned into 2 groups, namely group D (for treatment with dexmedetomidine) and group C (control). The randomization will be implemented with statistical software R, and stored in our database. Each patient will be registered with a unique ID. After the clinical assessment, patients will be assigned to surgeons and allocated into distinct treatment groups randomly. Dexmedetomidine and saline will be in the package with the same looking, whereas surgeons and patients will not be aware of the grouping.

### 2.13 Statistical analysis

Baseline characteristics of patients will be summarized by groups and compared using Chi-square or Fisher exact tests for categorical variables and 2-sample *t* tests or Wilcoxon rank sum test for the continuous variables. Continuous variables will be examined for normal distribution assumption. If there is a violation of distribution assumption, appropriate transformation will be used.

#### 2.13.1 Primary outcome (IL-6, TNF-α, and CRP in peripheral blood)

Analysis will be based on intention-to-treat principle. We will use repeated measurement ANCOVA with IL-6 at 6 and 24 hours after the end of operation as the outcome, groups and the main predictor adjusting for time, and IL-6 before induction. A random effect will be included to accommodate the correlation between the repeated measurements. A significant effect of group indicates that the IL-6 is different between groups after the operations. Similar analysis approach will be used for TNF-α and CRP in peripheral blood. A *P* value <0.05 will be considered as significant.

MMSE score will be compared using repeated measurement ANCOVA with MMSE score at 3 days, 6 days after the operation as the outcome variables, group as the main predictor, adjusting for the baseline MMSE score and time. A random intercept will be included to accommodate correlation between the repeated measurements.

#### 2.13.2 Secondary outcomes

Continuous outcome variables including heart rate (HR), systolic blood pressure (SBP), diastolic blood pressure (DBP), and ST segment changes in ECG will be analyzed in the following way. Descriptive statistical analysis will be performed with mean, SD by groups and time. Ninety-five percent confidence intervals will be calculated and 2-sample *t* tests will be used for comparison or median (inter quartile range) and Wilcoxon rank sum test will be used if normal distribution assumption is violated. Repeated measurement ANCOVA model will be used to compare them between groups during the follow-up period.

### 2.14 Adverse events

Adverse events will be analyzed as a binary variable. The number and percentage of patients with AE will be calculated and compared using Chi-square test.

### 2.15 Missing data

We will evaluate whether missingness of the primary outcome depends on group using Chi-square test. We will also evaluate the group effect using intention-to-treat and per protocol data set and compare the analysis results to evaluate robustness of our analytical results.

## 3 Discussion

The population of elderly people worldwide is increasing currently. Nearly 60% of the people aging 60 develops hypertension and even higher for people of 70-year old or older.^[25]^ With the development of the medical techniques, more surgical treatments are becoming available for elderly people. Consequently, the postoperative adverse effects on elderly are emerging increasingly and should be paid particular attentions to. For senile people with hypertension, the postoperative myocardial injury and POCD after anesthesia and surgery are among the most common and concerning problems that may cause great burdens to patients, their families, and the whole society.

POCD is getting attentions increasingly and mainly affects elderly people after certain medical treatments. Although the pathogenesis of POCD is still not clear, it has been reported that the increasing age, genetic background and pre-existing cognitive impairment may contribute to the raising of this disease.^[26]^ In addition, it was thought to be caused by cardiopulmonary bypass technique and anesthesia.^[25,27,28]^ Thus it might be presumed that the appropriate approach of anesthesia may help to reduce the incidence of POCD. Dexmedetomidine has been reported to be associated with lower rate of postoperative cognitive delirium than midazolam or propofol.^[29]^ In combination with the fact that it could inhibit sympathetic activity, stress response, and protect the brain, it shows potential advantages in the improvement of postoperative cognitive function.

The present study is designed to investigate the effect of the application of dexmedetomidine on postoperative myocardial injury and POCD, also to explore the association between inflammatory factors and postoperative cognitive function. With this study, we are expecting to find out an appropriate anesthesia method for elderly people with hypertension to alleviate the postoperative adverse effects caused by medical treatments.

## Author contributions

XD and WM designed this study together. XD and JY performed the statistical analysis and drafted the protocol. WM was responsible for the writing revision.

**Data curation:** Xuejiang Du, Jianshe Yu, Weidong Mi.

**Formal analysis:** Xuejiang Du, Jianshe Yu.

**Investigation:** Xuejiang Du.

**Methodology:** Xuejiang Du.

**Project administration:** Xuejiang Du, Weidong Mi.

**Writing – original draft:** Xuejiang Du, Jianshe Yu.

**Funding acquisition:** Weidong Mi.

**Writing – review and editing:** Weidong Mi.
